# Comparative FT-IR Spectroscopy Salivary Profiles of University Students: E‑Cigarette Users vs Nonusers

**DOI:** 10.1021/acsomega.5c10219

**Published:** 2025-12-18

**Authors:** Camila Lopes Ferreira, Emanuelly Caroline dos Santos Rocha, Yasmin Ferreira Azevedo dos Reis, Raphael Zanon Guimarães, Sara Maria Santos Dias da Silva, Rodrigo Teodoro Gomes de Paiva, Jean Patrick dos Santos Moraes, Laurita dos Santos, Luis Felipe das Chagas e Silva de Carvalho

**Affiliations:** † Health Science Post-Graduate Program, 67891 University of Taubaté-UNITAU, Taubaté 12020-270, SP, Brazil; ‡ Undergraduate, University of Taubaté-UNITAU, Taubaté 12020-270, SP, Brazil; § Postgraduate in Health Science Post-Graduate Program, University of Taubaté-UNITAU, Taubaté 12020-270, SP, Brazil; ∥ Universidade Brasil-UNIBRASIL, São Paulo 08230-030, SP, Brazil

## Abstract

This study investigated whether the use of e-cigarettes induces detectable alterations in salivary composition through Fourier-transform infrared spectroscopy (FT-IR). Saliva samples were collected from 60 young university students (30 e-cigarette users and 30 nonusers) at the University of Taubaté (Taubaté-SP). A total of 181 spectra were acquired in triplicate, resulting in 90 spectra for e-cigarette users and 91 for controls, because one control was measured in quadruplicate. Data were preprocessed by baseline correction, Savitzky–Golay smoothing, and vector normalization. Three spectral regions were examined (3050–2800 cm^–1^, 1720–1490 cm^–1^, and 1200–900 cm^–1^), and discriminatory analysis was performed using a Support Vector Machine (SVM) classifier with repeated 10-fold cross-validation. In the 3050–2800 cm^–1^ region, controls exhibited five peaks (3290, 2962, 2925, 2874, and 2853 cm^–1^), while e-cigarette users showed six, including a unique band at 3273 cm^–1^ and shifts at 3288 and 2851 cm^–1^. In the 1720–1490 cm^–1^ amide region, controls presented peaks at 1640 and 1546 cm^–1^, whereas users exhibited peaks at 1649 and 1544 cm^–1^, indicating possible protein alterations. This region yielded the best classification performance (accuracy ∼ 0.65; F1-score ∼ 0.72; AUC ∼ 0.73). In the 1200–900 cm^–1^ fingerprint region, both groups shared a coincident peak at 1077 cm^–1^ with only minor intensity differences. There was not a clear separation between cigarette user and nonsmokers groups by PCA analysis. Within the limitations of this study, the findings suggest that e-cigarette use may induce measurable biochemical changes in saliva, particularly in protein- and lipid-associated vibrational modes. These alterations could impair salivary defense functions, underscoring the importance of dental professionals in preventive counseling.

## Graphical abstract

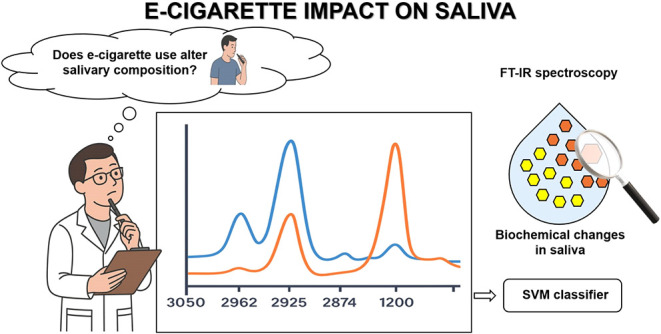

## Introduction

The use of electronic cigarettes (E-Cigarette), popularly known as “vapes,” has significantly increased, especially among young people. Presented as a potentially safer alternative to combat nicotine addiction, its growing use has raised concerns regarding the possible long-term adverse health effects.^1^

Several publications on the impact of electronic cigarettes on the oral cavity have pointed out adverse effects such as periodontal problems, dental caries, and reduced salivary flow.^2–6^ The vapor produced by electronic cigarettes, due to the chemical substances present in the liquid used, comes into direct contact with the oral cavity during inhalation, potentially causing significant impacts on oral health.^7^

Considering these findings, the need for a more detailed investigation into their effects is highlighted, both to promote improvements in public health and to increase awareness and advance knowledge in this area of study, especially considering the potential risk of developing oral diseases with the possibility of malignant transformation, such as cancer.^8–10^

Saliva plays essential roles in maintaining oral health, and changes in its composition can indicate imbalances and predispositions to various diseases.^11–13^ Taking this into consideration, Fourier Transform Infrared (FTIR) spectroscopy, a technique used to analyze the chemical structure and composition of materials through their interaction with infrared radiation, has been applied to human biofluids for the early diagnosis of various diseases. Among these biofluids, saliva stands out due to its easy accessibility and collection, as well as its great potential for utilization.^14^

Characterization of saliva constituents in the context of different pathological conditions allows for a better understanding of the pathophysiology involved in various situations, in addition to favoring the development of rapid, accessible, noninvasive, and clinically applicable diagnostic methods.^12,15,16^

This study aimed to analyze salivary alterations in electronic cigarette users, comparing them with nonsmokers. For this purpose, FTIR spectroscopy was employed to perform a comparative analysis, focusing on the effects of electronic cigarettes on the oral cavity and exploring possible associations between vaping and the incidence of oral diseases.

## Methodology

### Clinical Protocol and Patient Information

The present study was approved by the clinical research ethics committee under protocol number 19436919.7.0000.5501. The participants, volunteers among students at the University of Taubaté (Taubaté/SP), were informed about the research and signed an informed consent form before saliva sample collection.

The samples were divided into two groups: the first, composed of electronic cigarette users (T), and the second, a systemically healthy control group (S). “The T group was defined as individuals who use electronic cigarettes and do not abstain from conventional cigarettes or other substances. This information was obtained through an electronic self-report questionnaire, and it was not possible to classify the duration of prior consumption, dose dependence, or daily frequency of use within the collected sample.”

Saliva samples were collected from 60 young adults, comprising 30 e-cigarette users (group T) and 30 nonusers (group S). Each sample was analyzed in triplicate, resulting in 90 spectra for group T and 91 spectra for group S, as one control sample was acquired in quadruplicate.

### Sample Collection

Before saliva collection, patients were instructed to rinse their mouths with water. Collection was performed using the spitting method, where participants spat approximately 10 mL into universal collection vials. The saliva samples were transferred to microtubes and stored at −80 °C until analysis.

### Sample Processing

Samples were pipetted onto the spectrometer crystal. After each acquisition, the crystal was cleaned with 70% ethanol and allowed to dry completely before the next measurement. As the equipment features an ATR system with heating, which facilitates the drying process directly on the crystal. Spectra were also recorded after full sample drying. Raw spectral data in the 500–4000 cm^–1^ range were analyzed covering the high-wavenumber, amide region and fingerprint. To avoid contamination, a background scan was performed between each sample.

### Data Analysis

FT-IR spectra were acquired from two groups: 91 spectra from group S and 90 from group T. The spectral data were preprocessed in three steps. First, a linear baseline correction was applied using the ranges 4000–3735 cm^–1^ and 2329–1801 cm^–1^ as reference points. Second, spectra were smoothed using a Savitzky–Golay filter with a second-order polynomial and a window size of 11 points. Finally, each spectrum was vector-normalized (L2 norm) by dividing all intensity values by the Euclidean norm of the spectrum.

Three spectral regions of interest were analyzed: (a) 3050–2800 cm^–1^, (b) 1720–1490 cm^–1^, and (c) 1200–900 cm^–1^. Band positions were determined from local minima in the second derivative of the mean spectra. These spectral regions were selected based on their biochemical relevance to saliva composition, as they encompass vibrational modes associated with lipids (high-wavenumber CH stretching), proteins (amide I and II bands), and carbohydrate- and phosphate-related structures (fingerprint region). Preliminary tests using the full spectral range (500 −4000 cm^–1^) and the broader biofingerprint region (900 −1800 cm^–1^) were also performed; however, these approaches resulted in lower metrics values. Thus, focusing on the targeted regions improved the signal-to-noise ratio and enhanced the discriminatory capacity for the present data set. Band positions were determined from local minima in the second derivative of the mean spectra for each group.

To assess the discriminatory power of these regions, a Support Vector Machine (SVM) classifier with a radial basis function (RBF) kernel was implemented. Model performance was evaluated using 10-fold cross-validation repeated 20 times, ensuring robust estimates of predictive metrics. For each fold, approximately 8–10 samples from each group were included in the test set. The metrics calculated included accuracy, sensitivity (recall), specificity, precision, F1-score, and the area under the receiver operating characteristic curve (AUC)

accuracy=(TP+TN)/(TP+FN+TN+FP)

sensitivity(recall)=TP/(TP+FN)

specificity=TN/(TN+FP)

precision=TP/(TP+FP)

F1score=2×(precision×recall)/(precision+recall)

AUC = area under curve ROC TP = true positive; FN = false negative; TN = true negative; FP = false positive

The Support Vector Machines (SVM) was chosen as the primary classifier because it is one of the most widely used and well-validated machine learning methods for spectral data, particularly in small-to-moderate sample scenarios. SVM is known to perform robustly with high-dimensional, collinear data sets such as FT-IR spectra, and it has been successfully applied in several previous studies involving classification of biological or chemical samples.^17,18^

## Results

The FT-IR spectroscopic analysis of saliva samples from e-cigarette users (group T) and healthy controls (group S) generated raw spectral data within the 500–4000 cm^–1^ range (Figure 1).

**Figure 1:**
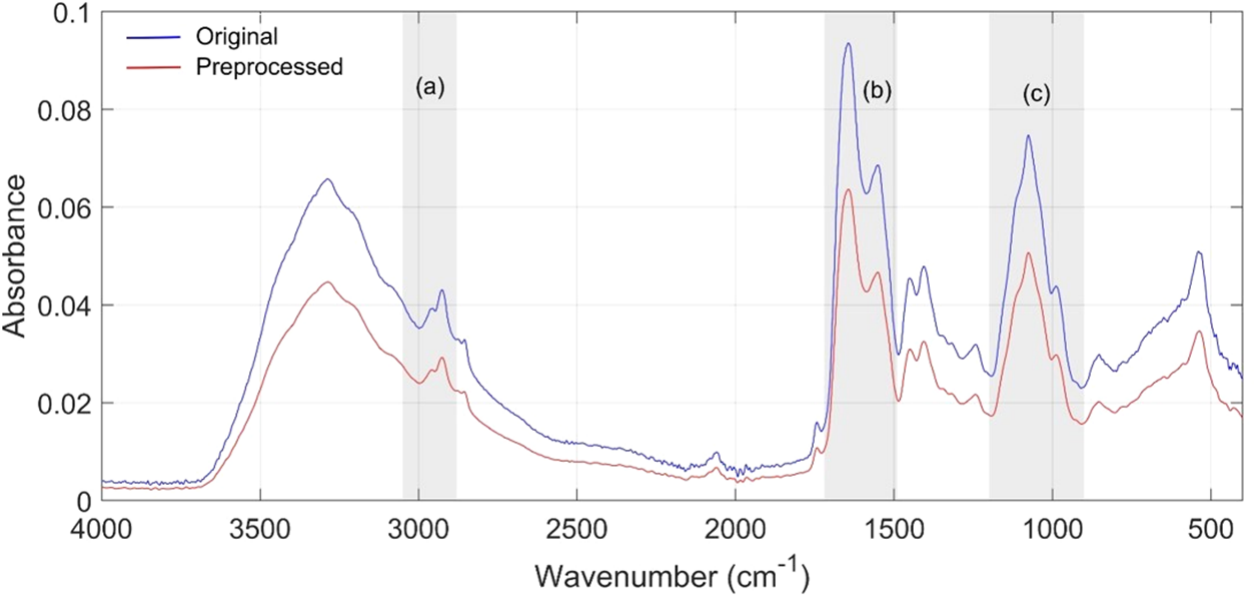
Preprocessing of an FTIR Spectrum Legend: Comparison between the original and preprocessed spectrum. Highlighted regions in gray: (a) 3050–2800 cm^–1^, (b) 1720–1490 cm^–1^, (c) 1200–900 cm^–1^.

In the high wavenumber region (3050–2800 cm^–1^), the mean salivary spectra of both groups revealed characteristic peaks corresponding to water, proteins, lipids, carbohydrates, and nucleic acids (Figure 2 and Table 1).

**Figure 2:**
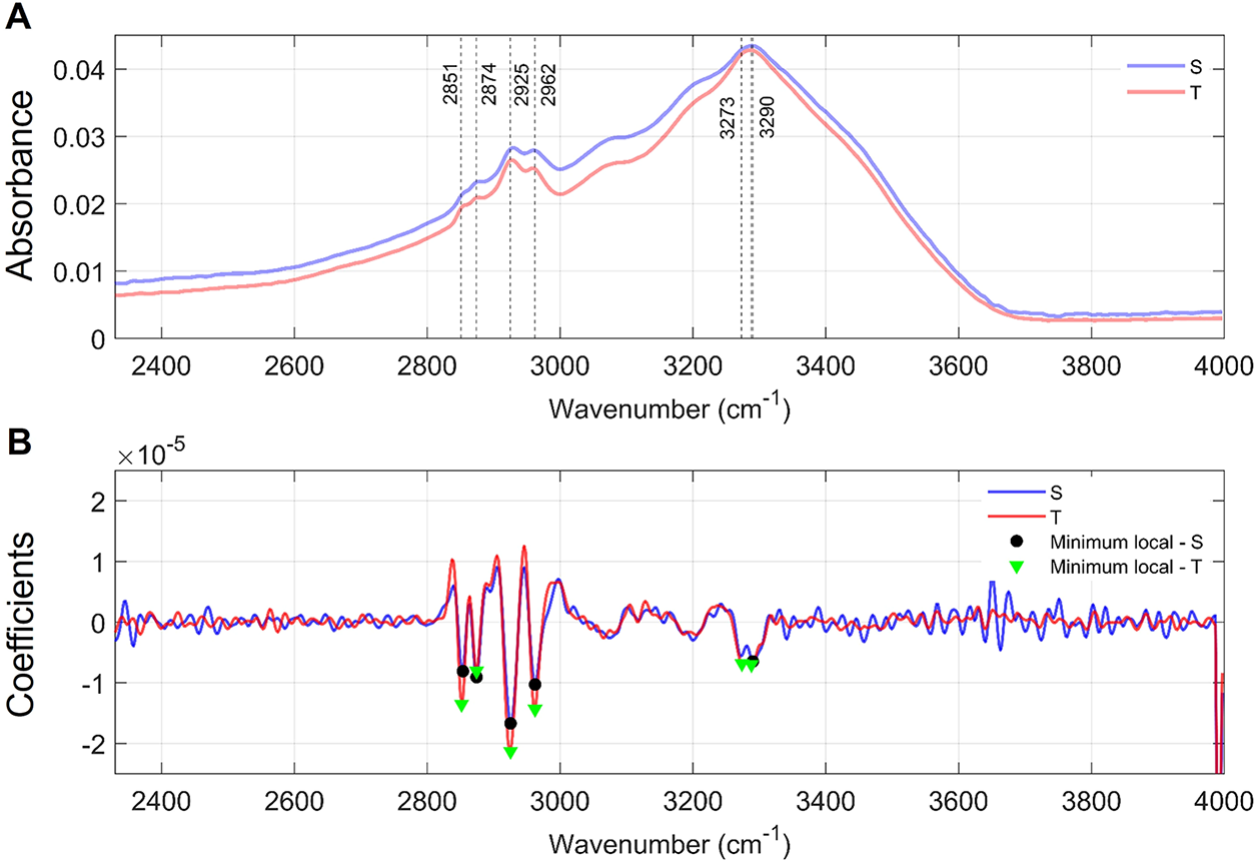
Comparison of the mean spectra for the high wavenumber region. Legend -(A) Mean spectra highlighting the band centers established from the local minima obtained from the second derivative of the mean spectra of each group (B). Note that there are five local minima for group S (black markers) and six local minima for group T (green markers). Peaks in group S occur at 3290 cm^–1^, 2962 cm^–1^, 2925 cm^–1^, 2874 cm^–1^, and 2853 cm^–1^, whereas peaks in group T occur at 3288 cm^–1^ (suggesting a shift), 3273 cm^–1^ (present only in group T), 2962 cm^–1^, 2925 cm^–1^, 2874 cm^–1^, and 2851 cm^–1^ (also suggesting a shift).

**Table 1:** Bands and Biomolecular Components^a^

bands (cm^–1^)	vibrational modes	biomolecular components	group where vibrational mode appears
3290 (3288)	stretching OH symmetric	water	both groups – maybe a shift 3288 cm^–1^ (T group)
3273	νO–H (water), νN–H (protein)	water and proteins	T group
2962	CH_3_ asymmetric stretching, CH stretching, ν_as_ CH_3_, asymmetric stretching mode of the methyl groups	lipids, proteins, carbohydrates and nucleic acids	both groups
2925	νC–H, ν_as_ CH_2_, and CH_3_, stretching C–H	lipids, proteins, carbohydrates and nucleic acids	both groups
2874	ν_s_ CH_3_, stretching C–H and N–H, CH_3_ symmetric stretching, symmetric stretching vibration of CH_3_ of acyl chains	lipids, peptides/proteins, and contribution from carbohydrates, nucleic acids with C–H and N–H bonds	both groups
2853 (2851)	ν_s_ CH_2_ of lipids, νC–H, CH_2_ symmetric stretching, asymmetric CH_2_ stretching mode of methylene chains in membrane lipids	lipids and small contribution of carbohydrates, nucleic acids and proteins with C–H bonds	both groups – maybe a shift 2851 cm^–1^ (T group)

a Legend: Based on Monteiro et al.^19^.

Group S presented five local minima at 3290, 2962, 2925, 2874, and 2853 cm^–1^, whereas group T exhibited six minima, including a unique band at 3273 cm^–1^ and slight shifts at 3288 and 2851 cm^–1^. These variations highlight subtle differences in band positions between groups, while most peaks were common to both. Table 1 summarizes the vibrational assignments of these bands, indicating their relation to stretching modes of groups, with contributions from water, proteins, lipids, carbohydrates, and nucleic acids.

In the 3050–2800 cm^–1^ region, group S exhibited five band centers at 3290, 2962, 2925, 2874, and 2853 cm^–1^, while group T presented six band centers, including a unique peak at 3273 cm^–1^ and slight shifts at 3288 and 2851 cm^–1^. These variations suggest alterations in lipid- and protein-associated vibrational modes. Classification with the SVM model achieved a mean accuracy of 0.6994 ± 0.0169, with very high sensitivity (1.0000 ± 0) but no specificity (0.0). The model also showed a precision of 0,4972 ± 0.0, an F1-score of 0.6642 ± 0.0, and an AUC of 0.7623 ± 0.0191 (Table 2).

**Table 2:** High Wavenumber Region 2800–3050 cm^–1^

metrics calculated from the SVM classification	mean value ± standard deviation (20 repetitions of 10-fold cross-validation)
accuracy	0.6994 ± 0.0169
sensitivity (recall)	1.0000 ± 0
specificity	0.0
F1 score	0.6642 ± 0.0
precision	0,4972 ± 0.0
AUC	0.7623 ± 0.0191

The ROC curve obtained from one of the 20 rounds of the SVM to show the AUC value for this wavenumber range (Figure 3).

**Figure 3:**
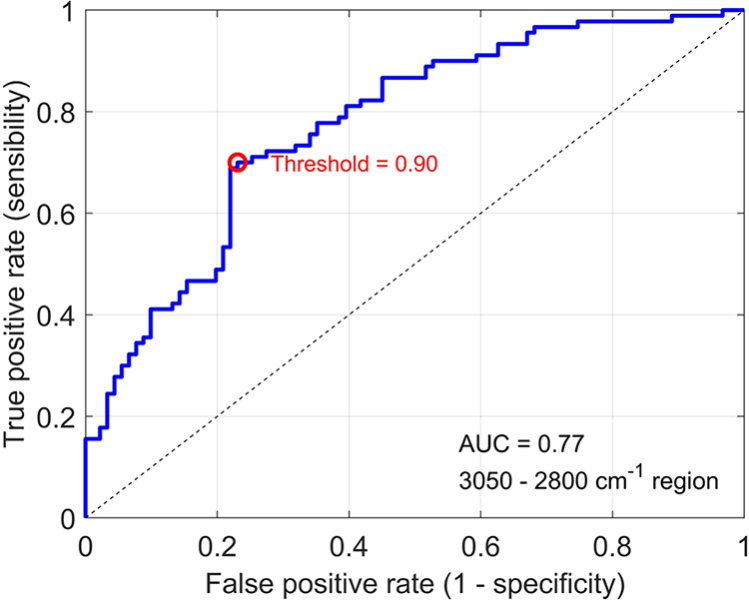
ROC curve of 2800–3050 cm^–1^ range.

In the amide region (1720–1490 cm^–1^), the mean spectra revealed two peaks for both groups. Group S presented band centers at 1640 and 1546 cm^–1^, while group T showed band centers at 1649 and 1544 cm^–1^, with small shifts in peak position and subtle differences in absorbance intensity (Figure 4).

**Figure 4:**
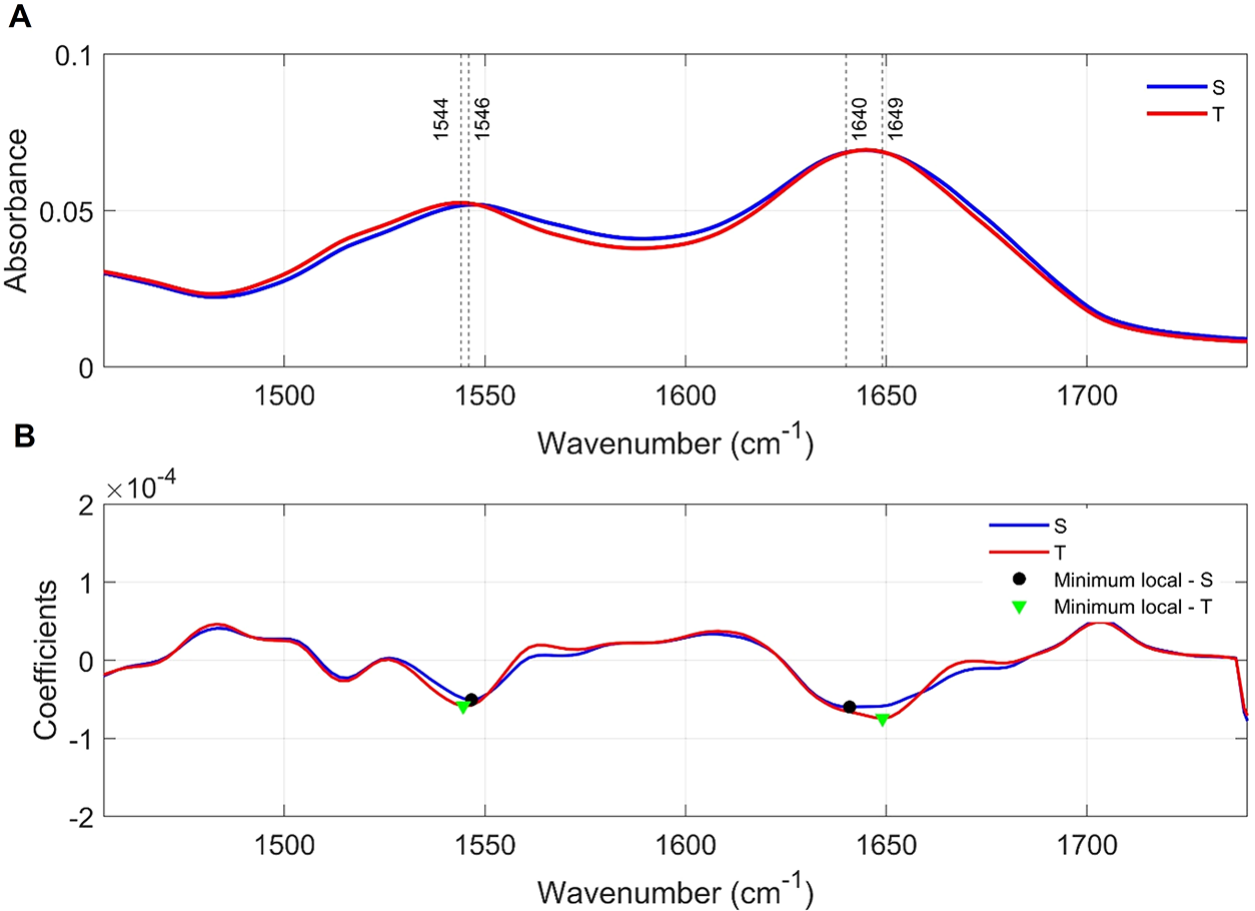
Amide Region. Comparison of the mean spectra in the 1720–1490 cm^–1^ range. Legend: (A) Mean spectra highlighting the band centers established from the local minima obtained from the second derivative of the mean spectra of each group in (B). Note that there are 2 local minima for both group S and group T. However, the absorbance intensity is slightly different for some mean band centers.

The vibrational assignments of these bands, mainly associated with amide I and amide II modes of proteins and peptides, are summarized in (Table 3).

**Table 3:** Vibrational Bands and Biomolecular Components in the Amide Region (1720–1490 cm^–1^)­^a^

bands (cm^–1^)	vibrational modes	biomolecular components	group where vibrational mode appears
1649	amide I (proteins/H–OH deformations of water, amide I)	proteins and water	T group
1640	α-helix	protein	S group
1546	amide II band (N–H stretch); α-helical structure	proteins and peptide	S group
1546 (1544)	amide II band, amide II band (N–H stretch), amide I (CN stretch and NH bend)	proteins and peptide	T group–maybe a shift in 1546 (S group)

a Legend: Based on Naseer et al.;^20^ Barth and Zscherp, 2002 ^21^ and Talari et al.^22^.

Classification using the SVM model in this region demonstrated better discriminatory performance compared with the high-wavenumber region, achieving a mean accuracy of 0.65 ± 0.01, sensitivity of 0.99 ± 0.00, specificity of 0.21 ± 0.01, precision of 0.55 ± 0.00, F1-score of 0.71 ± 0.00, and an AUC of 0.73 ± 0.02 (Table 4).

**Table 4:** SVM Metrics for 1720–1490 cm^–1^ region

SVM classification metrics	mean value ± standard deviation (20 repetitions of 10-fold cross-validation)
accuracy	0.6555 ± 0.0188
sensitivity (recall)	0.9994 ± 0.0025
specificity	0.2198 ± 0.0133
F1 score	0.7169 ± 0.0039
precision	0.5589 ± 0.0044
AUC	0.7348 ± 0.0262

The ROC curve obtained from one of the 20 rounds of the SVM illustrates the AUC value for this wavenumber range (Figure 5).

**Figure 5:**
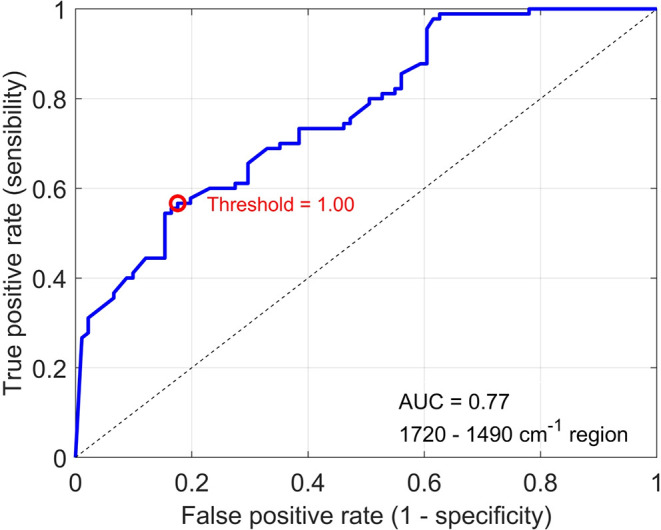
ROC curve of 1720–1490 cm^–1^ range.

In the fingerprint region (1200–900 cm^–1^), both groups exhibited a coincident band at 1077 cm^–1^, with minor differences in absorbance intensity between the groups (Figure 6).

**Figure 6:**
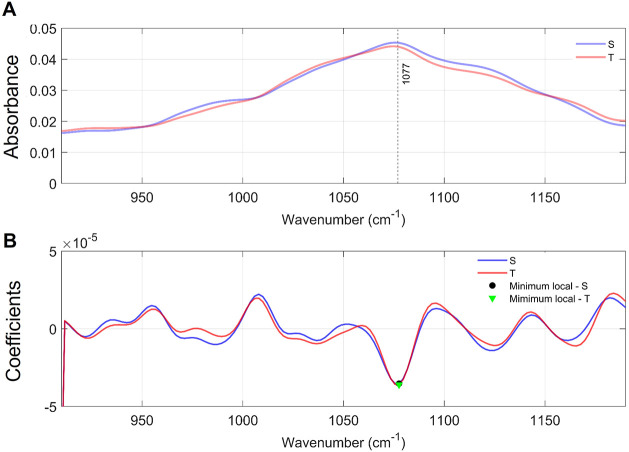
Fingerprint Region. Legend: (A) Comparison of the mean spectra in the 1200–900 cm^–1^ region. (B) Mean spectra highlighting the band centers established from the local minima obtained from the second derivative of the mean spectra of each group.

The vibrational assignment of this band, mainly related to sugar moieties, glycosylated proteins, phosphate groups, and glycogen, is summarized in Table 5.

**Table 5:** Vibrational Band and Its Biomolecular Assignments in the Fingerprint Region (1200–900 cm^–1^)­^a^

bands (cm^–1^)	vibrational modes	biomolecular components	group where vibrational mode appears
1077	sugar moieties and glycosylated proteins and PO_4_ compounds; skeletal *cis* conformation (CC) of DNA; symmetric phosphate [PO_2_^–^ (sym)] stretching; ns PO_2_^–^; phosphate I in RNA; symmetric phosphate; glycogen absorption due to CO and CC stretching and COH deformation motions; DNA in healthy samples, in the absence of glycogen; indicating the role of phosphates during diseases; COH stretching band of oligosaccharide residue	carbohydrates, phosphate groups (PO_2_^–^) and glycogen or other sugar derivatives	both groups
1125	CH2,6 in-plane bend and C1–Ca–Ha bend *n*(CO) *n*(CC) ring (polysaccharides, cellulose)	polysaccharides; structural carbohydrates	S group

a Legend: Based on Naseer et al.,^20^ Barth and Zscherp, 2002 ^21^ and Talari et al.^22^.

The SVM model yielded an accuracy of 0.62 ± 0.01, sensitivity of 0.99 ± 0.00, specificity of 0.12 ± 0.03, precision of 0.52 ± 0.00, F1-score of 0.69 ± 0.00, and an AUC of 0.68 ± 0.02 (Table 6).

**Table 6:** SVM Metrics for 1200–900 cm^–1^Region

SVM classification metrics	mean value ± standard deviation (20 repetitions of 10-fold cross-validation)
accuracy	0.6204 ± 0.0160
sensitivity (recall)	0.9978 ± 0.0046
specificity	0.1236 ± 0.0356
F1 score	0.6921 ± 0.0081
precision	0.5298 ± 0.0096
AUC	0.6897 ± 0.0227

The ROC curve obtained from one of the 20 rounds of the SVM illustrates the AUC value of the fingerprint region (Figure 7).

**Figure 7:**
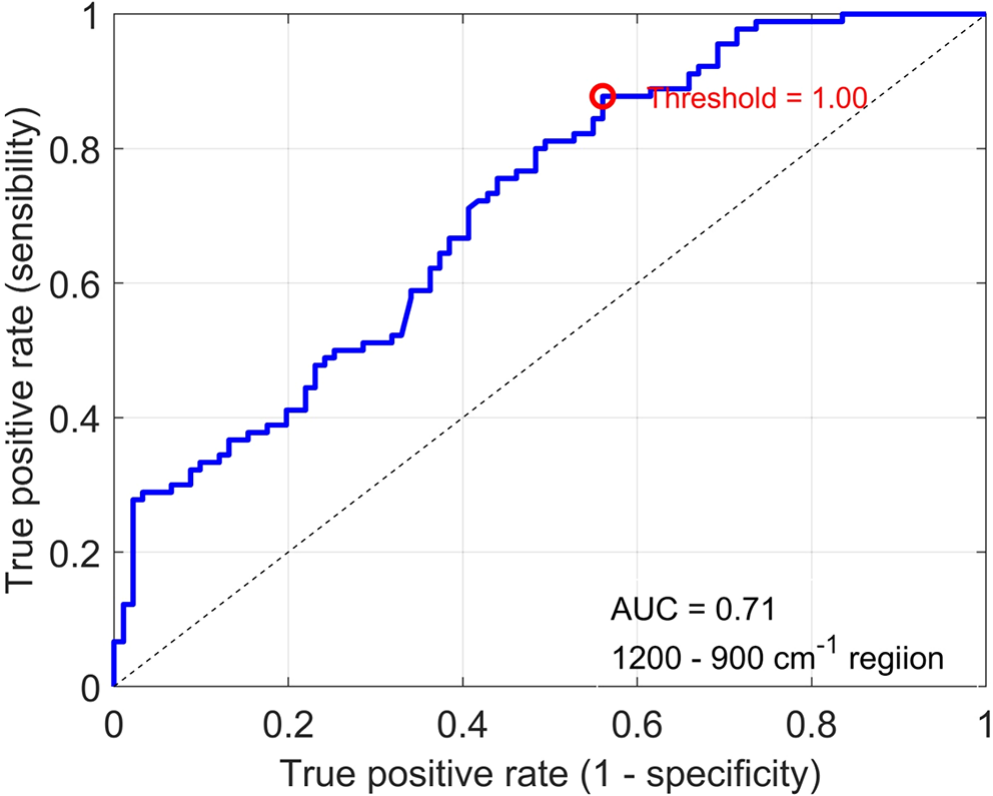
ROC curve of 1200–900 cm^–1^ range.

The Principal Component Analysis (PCA) did not show a clear separation between e-cigarette users and controls in either two- or three-dimensional space (Figure 8).

**Figure 8:**
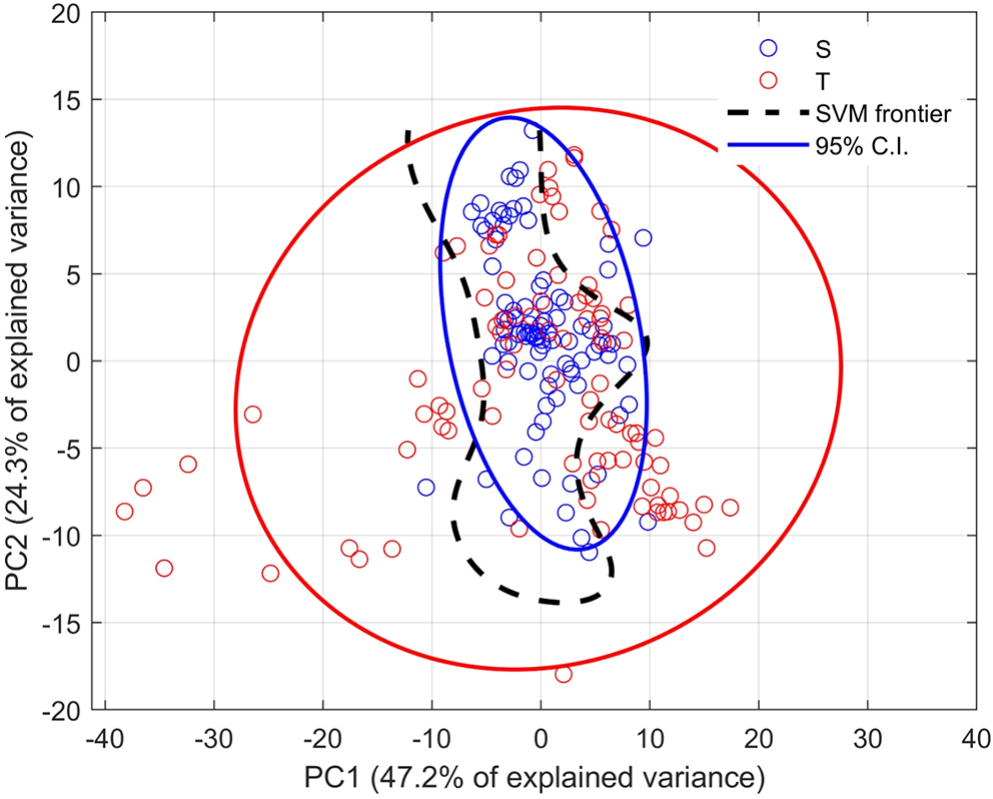
SVM visualization on PCA space. Legend: SVM decision boundary plotted over the 2D PCA projection of FTIR spectral data. Blue circles correspond to the control (nonsmoker) group (S), red circles to the smoker group (T). The dashed black line indicates the SVM decision frontier, and the ellipses represent 95% confidence intervals for each group.

Although some spectral differences were detected, clustering overlap indicated limited discriminatory capacity, consistent with the moderate sensitivity and specificity values observed in the classification analyses. This was confirmed by the sensitivity and specificity matrix (SESM), which showed percentage values that were not sufficiently high to generate robust discrimination between groups.

To provide a visual interpretation of the classification model, Figure 8 shows the two-dimensional projection of the FTIR spectral data onto the first two principal components (PC1 = 47.2% of explained variance, PC2 = 24.3% of explained variance) with the SVM decision boundary superimposed. Although the SVM classifier operates in a higher-dimensional kernel space, this projection enables qualitative assessment of the group distribution. The blue and red ellipses represent the 95% confidence intervals (C.I.) of the S and T groups, respectively. The dashed black line indicates the SVM decision frontier derived from the classification model. As shown, there is partial overlap between the two groups, consistent with the biological similarity of salivary biochemical composition. Nonetheless, the SVM boundary correctly separates the central tendencies of both groups, corroborating the discriminative patterns captured by the model. This visualization supports the statistical findings (accuracy, sensitivity, specificity) and provides additional qualitative evidence of the SVM model’s ability to distinguish smokers from nonsmokers based on FTIR spectral features.

## Discussion

The use of electronic cigarettes, popularly known as “vapes,” has significantly increased, especially among young people, who often perceive it as a less harmful alternative to traditional cigarettes.^1^ Marketed with a modern image and as a potential tool for reducing nicotine addiction, vaping carries the promise of less harm to health compared to conventional tobacco.^23^

Previous studies have shown that the use of tobacco products, including traditional and electronic cigarettes, is associated with alterations in saliva composition and overall oral health.^24,25^ It was demonstrated that saliva can be used as a diagnostic medium for conditions such as diabetes and periodontal diseases, corroborating the idea that saliva reflects significant biochemical changes in the body.^26^ Furthermore, the literature suggests that electronic cigarette use may be correlated with an increase in inflammatory markers, reinforcing the relationship between systemic and oral health.^27^

In the context of this growing evidence, our study contributes by providing FT-IR-based biochemical characterization of saliva from electronic cigarette users, revealing subtle but measurable spectral alterations across lipid-, protein-, and carbohydrate-associated vibrational modes. These results offer objective spectroscopic support to the hypothesis that e-cigarette exposure influences salivary composition, even in young, systemically healthy individuals.

In the present study, FT-IR analysis of saliva samples from e-cigarette users and controls revealed differences in specific spectral regions. In the high-wavenumber region (3050–2800 cm^–1^), e-cigarette users showed an additional band at 3273 cm^–1^ and slight shifts at 3288 and 2851 cm^–1^ compared with controls. In the amide region (1720–1490 cm^–1^), both groups exhibited two band centers, with controls showing bands at 1640 and 1546 cm^–1^ and users presenting bands at 1649 and 1544 cm^–1^, suggesting subtle modifications in protein-related vibrational modes. In the fingerprint region (1200–900 cm^–1^), both groups presented a coincident peak at 1077 cm^–1^, with only minor intensity differences. Peaks present in both groups suggest that some fundamental salivary components remain preserved; however, variations in their intensity may indicate differences in concentration or stability, influenced by e-cigarette exposure.

The band shifts observed in the 3050–2800 cm^–1^ interval are compatible with alterations in vibrational modes associated with lipids and proteins, indicating potential changes in membrane-derived components and salivary protein conformation. Small variations in CH_2_/CH_3_ stretching intensities have been previously linked to modifications in lipid packing, increased epithelial desquamation, or oxidative processes—mechanisms that have also been reported in FT-IR studies assessing vaping-related biochemical changes.^28^

Similarly, the slight displacement of amide I and II bands (1640 → 1649 cm^–1^ and 1546 → 1544 cm^–1^) suggests changes in the secondary structure of salivary proteins. Shifts in these regions are commonly associated with alterations in α-helix and β-sheet content, protein unfolding, or increased interaction with exogenous compounds. Prior studies have indicated that exposure to e-cigarette aerosol can affect protein integrity, oxidative balance, and microbial interactions in the oral cavity,^24,25^ supporting the hypothesis that protein modifications detected by FT-IR may reflect early biochemical responses to aerosolized chemicals.

In the fingerprint region, the shared peak at 1077 cm^–1^—associated with sugar moieties, glycosylated proteins, and phosphate-containing compounds—indicates that major carbohydrate-rich structures such as mucins remain present in both groups. However, minor intensity differences may reflect subtle alterations in mucin concentration or glycosylation, which are essential for maintaining lubrication, buffering capacity, microbial control, and epithelial protection. Such changes align with recent evidence showing that vaping can impact mucosal defenses and modify the oral microbiome.^27,28^

These results indicate a potential degradation of the natural defense capacity of saliva, which may increase the predisposition to inflammation and oral infections. The findings of this study have important implications for public health and clinical practice. As electronic cigarette use becomes increasingly common, it is crucial to understand the consequences of its use on oral health. The fact that alterations in saliva can impair the body’s natural defenses and increase the risk of oral infections highlights the urgency of educational and preventive measures directed at electronic cigarette users.^29^ Saliva, as an accessible biofluid, can serve as a noninvasive indicator of oral health, enabling early diagnoses and interventions before more severe problems develop.

The PCA analysis did not show clear group separation, indicating that although spectral differences were present, they were not sufficient to discriminate groups with high sensitivity and specificity. This finding is consistent with the SVM classification results, which demonstrated high sensitivity but only moderate specificity across the analyzed spectral regions. This suggests that, while spectral alterations are detectable, their discriminatory power may be limited without larger data sets or complementary analytical approaches.

While the findings presented here are promising, some methodological constraints should be acknowledged. The limited sample size did not permit the inclusion of an external validation set, leading us to rely exclusively on k-fold cross-validation. Although this approach is commonly adopted in spectroscopic studies with restricted data sets, it does not fully ensure external generalizability and may increase susceptibility to overfitting. Accordingly, the reported performance metrics should be interpreted with caution. Future studies involving larger cohorts and independent test sets will be essential to further substantiate and extend the present results. Furthermore, the PCA analysis revealed that, despite the observed differences, the sensitivity and specificity of the data were not sufficiently high to be clinically relevant.

This inconclusiveness may be attributed to a variety of factors, including the natural biological variability among individuals, the possible presence of other comorbidities that were not controlled for in this study, and the complexity of biochemical interactions in saliva. Additionally, the duration of exposure and the frequency of electronic cigarette use by the participants were not rigorously controlled, which could influence the results.

A methodological consideration involves machine learning analysis. Although the SVM classifier demonstrated high sensitivity across regions, specificity remained low, indicating some degree of overlap between the spectral profiles of users and controls. This behavior is not unexpected in studies involving biofluids with naturally high interindividual variability and subtle biochemical differences. It also highlights that triplicate spectral acquisition increases the number of spectra but not the number of independent biological samples, a factor that should be considered when interpreting classification performance.

To advance the understanding of the effects of electronic cigarette use on oral health, future research should include a larger number of participants, encompassing different demographics and consumption behaviors. Furthermore, longitudinal studies that monitor changes in saliva over time in electronic cigarette users could provide valuable insights into the progression of biochemical alterations. Investigating the relationship between assorted brands and compositions of electronic cigarette liquids and their respective consequences on oral health may also be a fruitful area for research.

Finally, the exploration of educational and preventive interventions aimed at electronic cigarette users may be crucial in mitigating the negative impacts on oral and overall health. Dental professionals should be attentive to these risks and proactive in counseling patients about the potential oral and systemic consequences of e-cigarette use.

## Conclusion

Fourier-Transform Infrared (FT-IR) spectroscopic analysis revealed notable alterations in the salivary composition of e-cigarette users, particularly in vibrational modes associated with proteins and lipids, compared with nonusers. These spectral differences, observed across the high-wavenumber, amide, and fingerprint regions were subtle but measurable, suggesting early biochemical changes that may reflect initial disturbances in oral homeostasis related to e-cigarette exposure. Although modest in magnitude, the shifts identified through FT-IR demonstrate the technique’s ability to detect salivary alterations of potential clinical interest. Within the limitations of the sample size and classification performance, these findings support the use of saliva as a noninvasive medium for monitoring vaping-related biochemical changes and underscore the necessity for targeted public health initiatives to raise awareness about the potential risks of e-cigarette consumption.

## References

[1] National Center for Chronic Disease Prevention and Health Promotion (US) Office on Smoking and Health . E-Cigarette Use Among Youth and Young Adults: A Report of the Surgeon General [Internet] Centers for Disease Control and Prevention (US): Atlanta (GA); 2016 https://www.ncbi.nlm.nih.gov/books/NBK538680/. 30869850

[2] Rouabhia M. (2020). Impact of
Electronic Cigarettes on Oral Health: a Review. J. Can. Dent Assoc..

[3] Ganesan S. M., Dabdoub S. M., Nagaraja H. N. (2020). Adverse effects of electronic
cigarettes on the disease-naive oral microbiome. Sci. Adv..

[4] Catala-Valentin A., Bernard J. N., Caldwell M., Maxson J., Moore S. D., Andl C. D. (2022). E-Cigarette Aerosol
Exposure Favors the Growth and
Colonization of Oral Streptococcus mutans Compared to Commensal Streptococci. Microbiol. Spectrum.

[5] Chaffee B. W., Halpern-Felsher B., Cheng J. (2023). E-cigarette, cannabis and combustible
tobacco use: associations with xerostomia among California adolescents. Commun. Dent. Oral Epidemiol..

[6] Shabil M., Khatib M. N., Ballal S. (2024). The impact of electronic
cigarette use on periodontitis and periodontal outcomes: a systematic
review and meta-analysis. BMC Oral Health.

[7] Famele M., Ferranti C., Abenavoli C., Palleschi L., Mancinelli R., Draisci R. (2015). The Chemical Components of Electronic
Cigarette Cartridges and Refill Fluids: Review of Analytical Methods. Nicotine Tob. Res..

[8] Ralho A., Coelho A., Ribeiro M., Paula A., Amaro I., Sousa J., Marto C., Ferreira M., Carrilho E. (2019). Effects of
Electronic Cigarettes on Oral Cavity: A Systematic Review. J. Evidence Based Dent. Pract..

[9] Auschwitz E., Almeda J., Andl C. D. (2023). Mechanisms
of E-Cigarette Vape-Induced
Epithelial Cell Damage. Cells.

[10] Gallagher K. P., Vargas P. A., Santos-Silva A. R. (2024). The use
of E-cigarettes as a risk
factor for oral potentially malignant disorders and oral cancer: a
rapid review of clinical evidence. Med. Oral
Patol. Oral Cir. Bucal.

[11] Meleti M., Cassi D., Vescovi P., Setti G., Pertinhez T. A., Pezzi M. E. (2020). Salivary biomarkers
for diagnosis of systemic diseases
and malignant tumors. A systematic review. Med.
Oral Patol. Oral Cir. Bucal.

[12] Boroumand M., Olianas A., Cabras T. (2021). Saliva, a bodily fluid
with recognized and potential diagnostic applications. J. Sep. Sci..

[13] do
Carmo Carvalho B. F., de Carvalho Faria N., Foiani L. (2024). Oral Mucosa
and Saliva Alterations Related to Vape. Clin.
Exp. Dent. Res..

[14] Delrue C., De Bruyne S., Speeckaert M. M. (2023). Unlocking the Diagnostic Potential
of Saliva: A Comprehensive Review of Infrared Spectroscopy and Its
Applications in Salivary Analysis. J. Pers.
Med..

[15] Nonaka T., Wong D. T. W. (2022). Saliva Diagnostics. Annu. Rev.
Anal. Chem..

[16] Song M., Bai H., Zhang P., Zhou X., Ying B. (2023). Promising applications
of human-derived saliva biomarker testing in clinical diagnostics. Int. J. Oral Sci..

[17] Tian P., Zhang W., Zhao H., Lei Y., Cui L., Wang W., Li Q., Zhu Q., Zhang Y., Xu Z. (2015). Intraoperative diagnosis of benign and malignant breast tissues by
fourier transform infrared spectroscopy and support vector machine
classification. Int. J. Clin. Exp. Med..

[18] Caixeta D.
C., Carneiro M. G., Rodrigues R., Alves D. C. T., Goulart L. R., Cunha T. M., Espindola F. S., Vitorino R., Sabino-Silva R. (2023). Salivary ATR-FTIR
Spectroscopy Coupled with Support Vector Machine Classification for
Screening of Type 2 Diabetes Mellitus. Diagnostics.

[19] Monteiro G. R., da Silva S. M. S. D., Rizzato J. M. B., de Lima Silva S., Cortelli S. C., Silva R. A., Nogueira M. S., das Chagas
e Silva de Carvalho L. F. (2024). High-Wavenumber Infrared Spectroscopy
of Blood Plasma for Pre-Eclampsia Detection with Machine Learning. Photonics.

[20] Naseer K., Ali S., Qazi J. (2021). ATR-FTIR spectroscopy
as the future of diagnostics:
a systematic review of the approach using bio-fluids. Appl. Spectrosc. Rev..

[21] Barth A., Zscherp C. (2002). What vibrations tell
us about proteins. Q. Rev. Biophys..

[22] Talari A. C. S., Martinez M. A. G., Movasaghi Z., Rehman S., Rehman I. U. (2017). Advances in Fourier transform infrared
(FTIR) spectroscopy of biological tissues. Appl.
Spectrosc. Rev..

[23] Palaia G., Mohsen M., Pergolini D. (2025). E-cigarette: a safe
tool or a risk factor for oral cancer? A systematic review. J. Clin. Exp. Dent..

[24] Ye D., Gajendra S., Lawyer G. (2020). Inflammatory biomarkers
and growth factors in saliva and gingival crevicular fluid of e-cigarette
users, cigarette smokers, and dual smokers: A pilot study. J. Periodontol..

[25] Kamal N. M., Shams N. S. (2022). The impact of tobacco
smoking and electronic cigarette
vaping on salivary biomarkers. A comparative study. Saudi Dent. J..

[26] Nogueira M. S., Barreto A. L., Furukawa M. (2022). FTIR spectroscopy as
a point of care diagnostic tool for diabetes and periodontitis: A
saliva analysis approach. Photodiagn. Photodyn.
Ther..

[27] Park B., Koh H., Patatanian M. (2023). The mediating roles of the oral microbiome
in saliva and subgingival sites between e-cigarette smoking and gingival
inflammation. BMC Microbiol..

[28] do
Carmo Carvalho B. F., Foiani L., Zucco G., Faria N. C., Nepomuceno G., Silva K. C. S., Borges R., Alves M. G. O., Pérez-Sayáns M., Martinho H. D. S., Almeida J. D. (2025). Decoding
E-Cigarette Secrets: Unveiling Saliva and E-Liquid Composition through
Fourier-Transform Infrared Spectroscopy. ACS
Omega.

[29] Effah F., Taiwo B., Baines D., Bailey A., Marczylo T. (2022). Pulmonary
effects of e-liquid flavors: a systematic review. J. Toxicol. Environ. Health, Part B.

